# Supplementary material for: Amplifying metabolic profiling of extracellular vesicle dynamics with ACTIVITY
Source: Nat Commun. 2026 Mar 26;17:4490. doi: 10.1038/s41467-026-71030-w (PMC13187049; doi:10.1038/s41467-026-71030-w)
Supplement: Supplementary file 1 — Supplementary Information [file 41467_2026_71030_MOESM1_ESM.pdf]

Supplementary Information for

Amplifying metabolic profiling of extracellular vesicle dynamics with ACTIVITY

Ru-Jia Yu,[1] Wei-Yi Ma,[1] Han-Yang Xiao,[1] Ya-Wei Zhang,[2] Wen-Bin Gong,[2] Zhen-Fei Yu,[1] Kai-Liang Wei, [1] Kuo-Ran Xing,[3] Xu Wang,[4] Hou-Juan Zhu,[5] Lian-Hui Wang,[1] Xian-Guang Ding[1,*]

[1] State Key Laboratory of Organic Electronics and Information Displays & Jiangsu Key Laboratory for Biosensors, Institute of Advanced Materials (IAM), Nanjing University of Posts and Telecommunications, Nanjing 210023, China
[2] School of Physics and Energy, Xuzhou University of Technology, Xuzhou 221018, China
[3] Department of Chemical and Biomolecular Engineering, National University of Singapore, Singapore 117585, Singapore
[4] The First Affiliated Hospital of Soochow University, Department of Central Intensive Care Unit of Anesthesiology, Suzhou 215006, China
[5] A*STAR (Agency for Science, Technology and Research), Singapore 138634, Singapore
* Xian-Guang Ding
Email: iamxgding@njupt.edu.cn

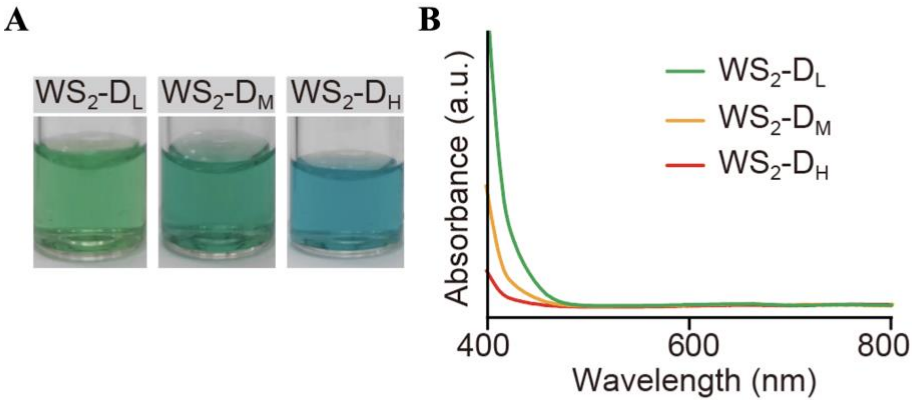

Supplementary Fig. 1 Stable colloidal suspension appearance of the defective WS_2 QDs (A) and corresponding UV-Vis absorption spectra (B). Source data are provided as a source data file.

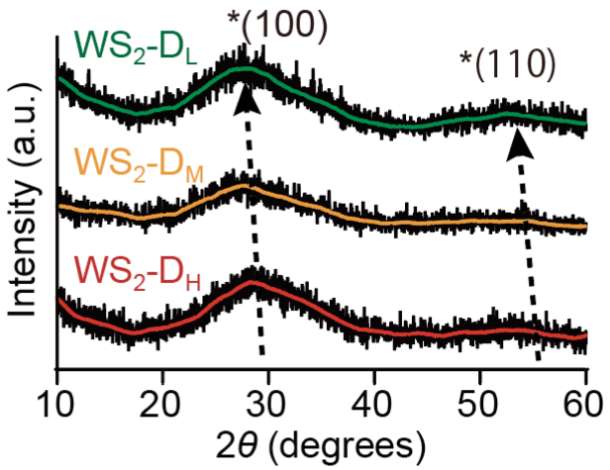

Supplementary Fig. 2 XRD spectra of these three kinds of defective WS_2 QDs. Source data are provided as a source data file.

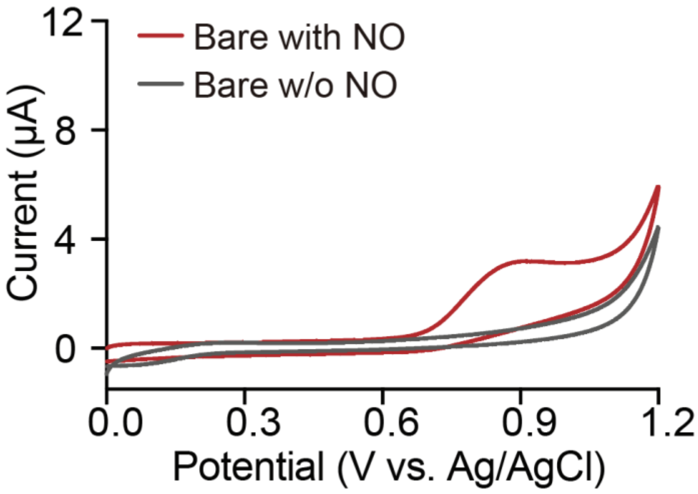

Supplementary Fig. 3 Cyclic voltammograms of bare glassy carbon electrodes in PBS electrolyte with or without 0.18 mM NO. Source data are provided as a source data file.

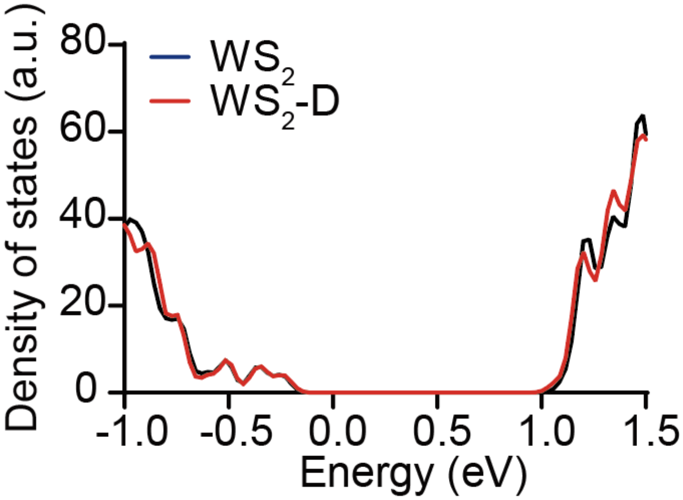

Supplementary Fig. 4 Calculated density of states of WS_2 and detective WS_2 QDs. Source data are provided as a source data file.

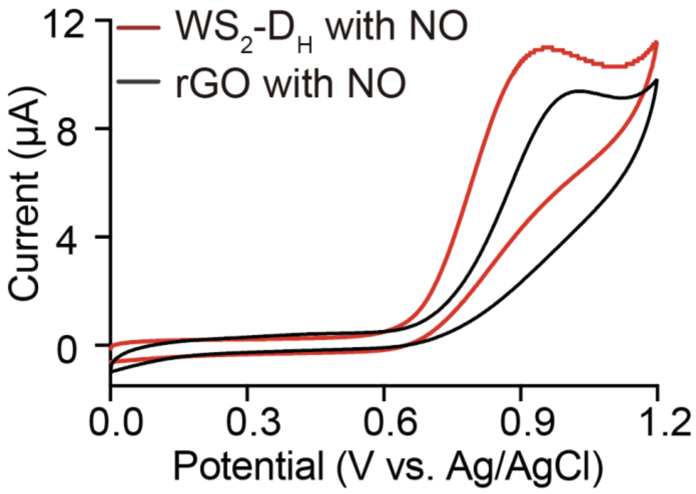

Supplementary Fig. 5 Cyclic voltammograms of WS_2 QDs (highly defective) and rGO modified electrodes in PBS electrolyte with 0.18 mM NO. Source data are provided as a source data file.

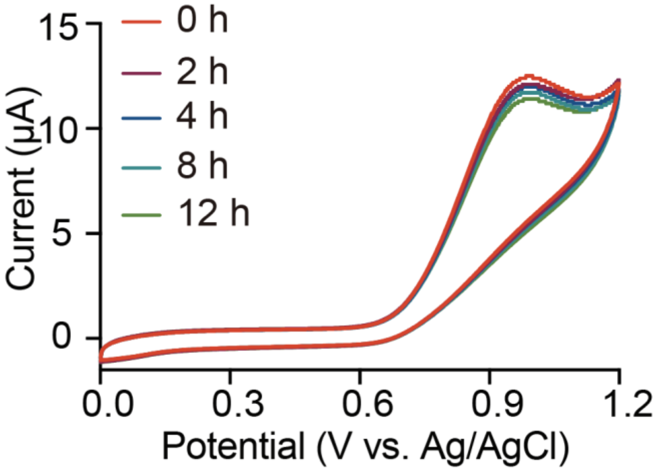

Supplementary Fig. 6 CV measurements of the WS_2-DH QDs modified electrode in PBS electrolyte with 0.18 mM NO over a 12-hour period, recorded at 2-hour intervals. Source data are provided as a source data file.

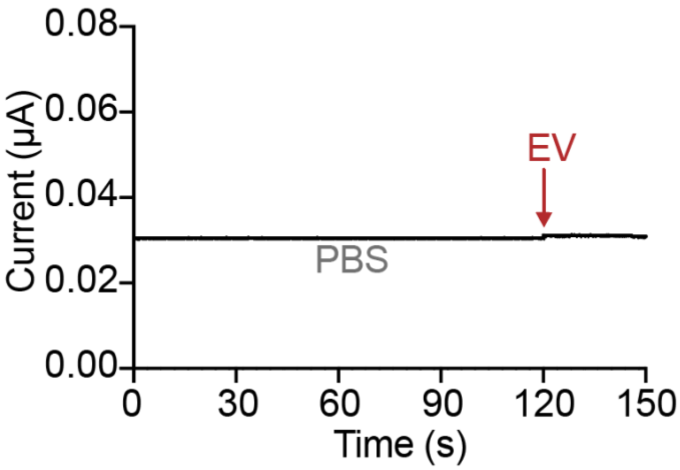

Supplementary Fig. 7 Amperometric response of the ACTIVITY method toward interference from EV lysates without the addition of enzyme substrate. Source data are provided as a source data file.

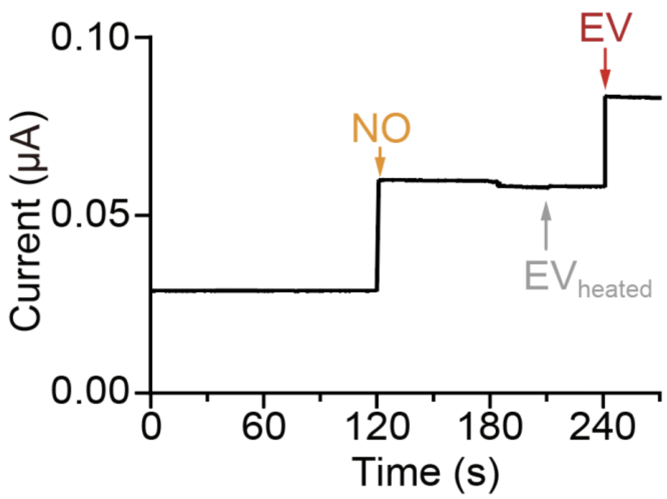

Supplementary Fig. 8 Amperometric response of the ACTIVITY method toward the EVs with (EV) and without (EV_{heated}) iNOS activity. Source data are provided as a source data file.

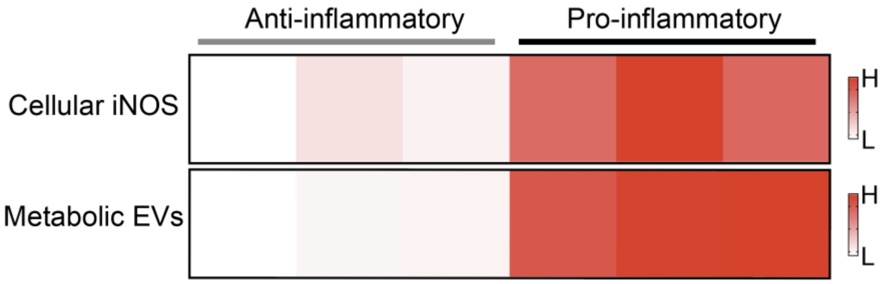

Supplementary Fig. 9 Relative current signals of the iNOS level in cell lysates (top) and metabolic EVs in supernatant (bottom). Source data are provided as a source data file.

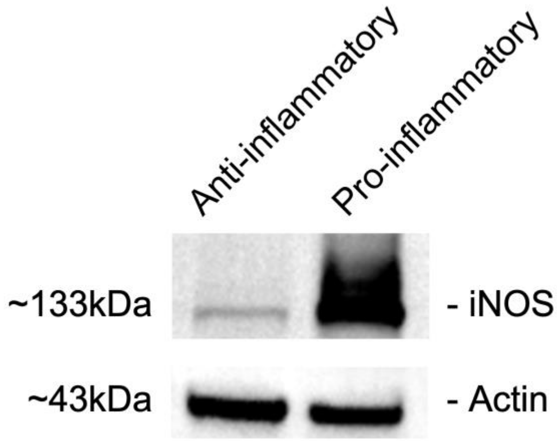

Supplementary Fig. 10 Western blot analysis of different polarized RAW264.7 macrophages. Source data are provided as a source data file.

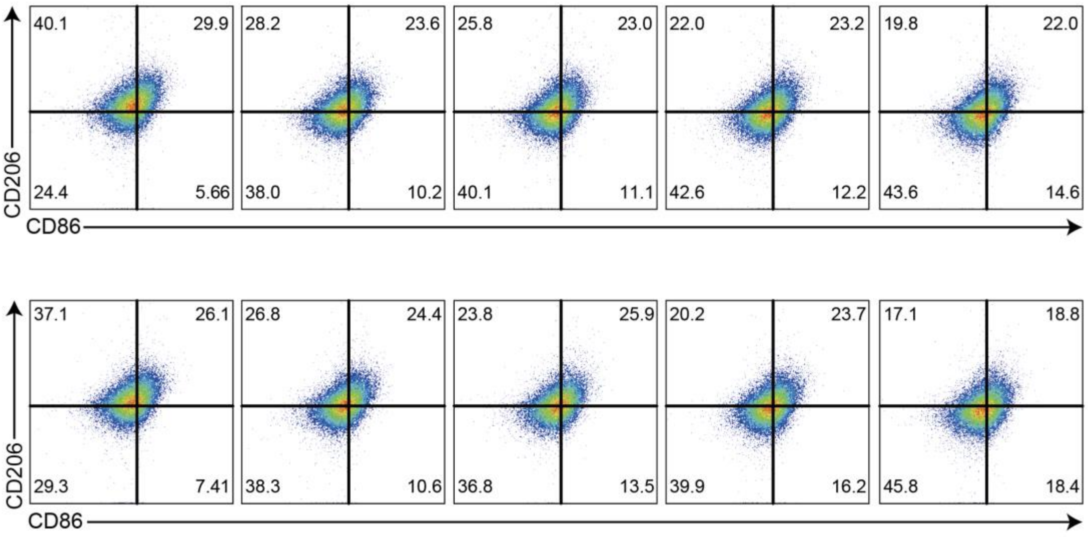

Supplementary Fig. 11 Flow cytometry analysis of CD86 and CD206 of macrophages incubated with LPS (top) and BLZ945 (bottom) for different periods at 0 h, 4 h, 8 h, 12 h and 24 h, respectively.

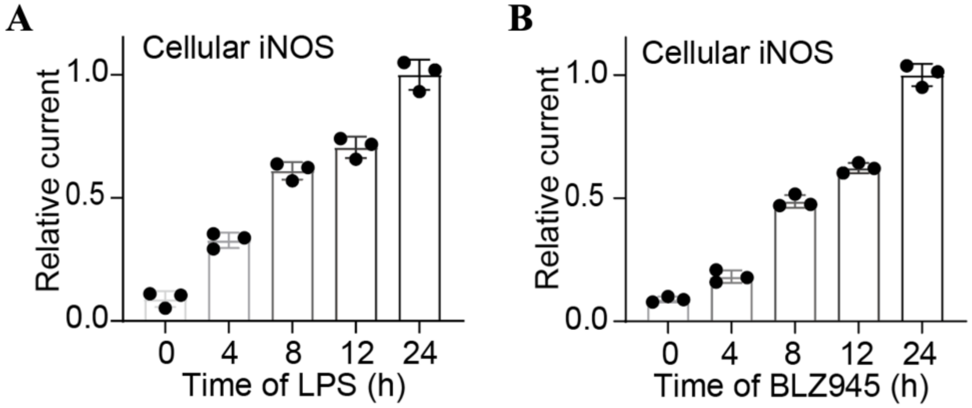

Supplementary Fig. 12 Electrochemical readout of iNOS levels in cell lysates by incubating macrophages with LPS (A) and BLZ945 (B) over time (n=3 independent experiments). Data are presented as mean ± SD. Source data are provided as a source data file.

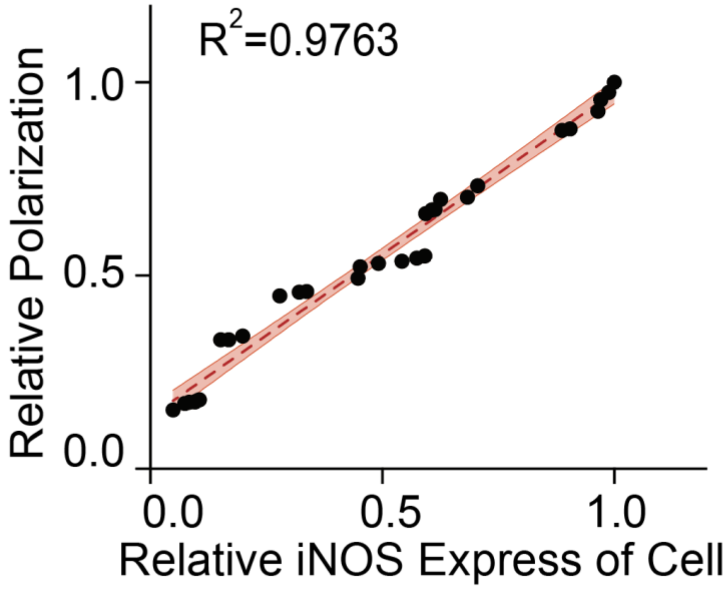

Supplementary Fig. 13 Correlation between macrophage polarization obtained from FCM results and relative expression of iNOS contents in cells. r=0.9881. Source data are provided as a source data file.

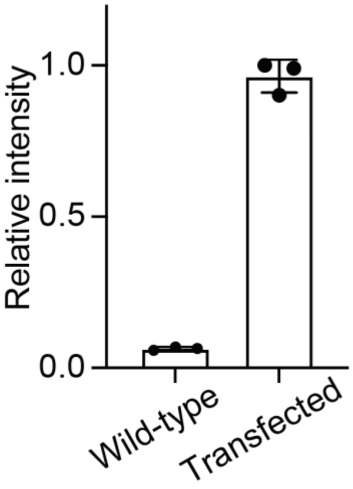

Supplementary Fig. 14 Fluorescence intensity of the GFP in wild-type and the transfected RAW 264.7 cells (n=3 independent experiments). Data are presented as mean ± SD. Source data are provided as a source data file.

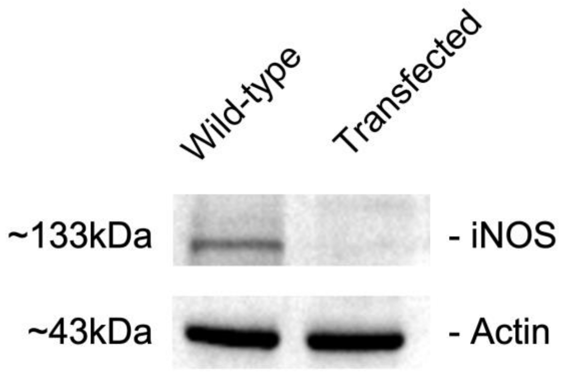

Supplementary Fig. 15 Western blot analysis of wild-type RAW264.7 macrophages and those transfected for iNOS knockout. Source data are provided as a source data file.

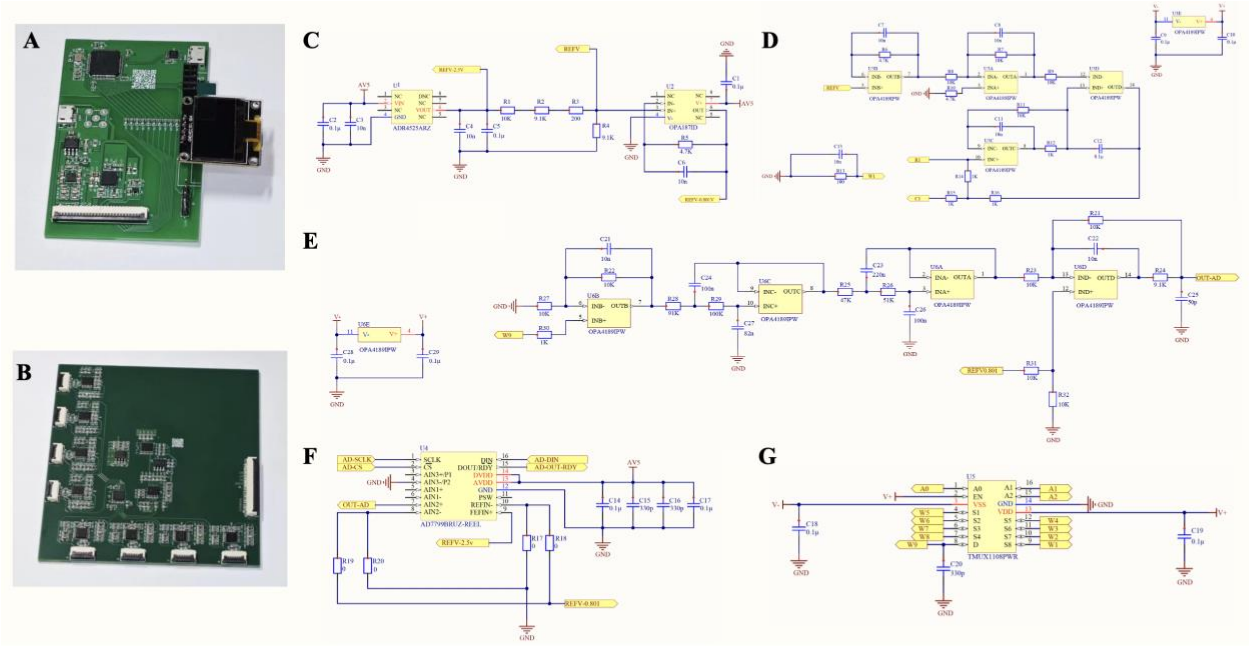

Supplementary Fig. 16 (A, B) Printed circuit boards for the development of the portable electrochemical device. (C-G) Partial circuit diagrams and component information for the electrochemical control and detection module.

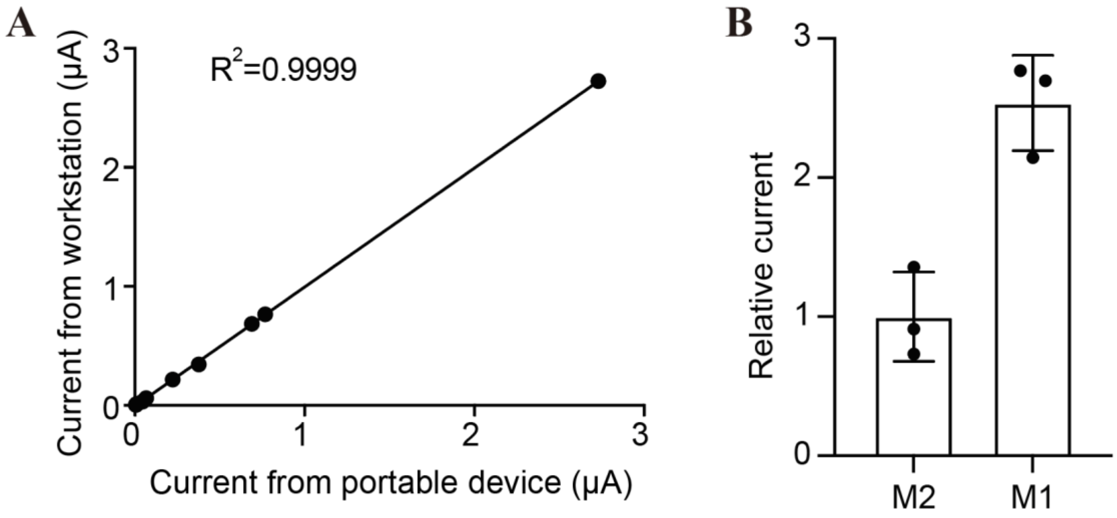

Supplementary Fig. 17 (A) The electrochemical detection performance of the home-designed miniaturized device compared with a commercial electrochemical workstation. It shows highly consistent results (R^2 = 0.9999) for NO detection across 11 concentrations ranging from 1.8 nM to 360 μM. (B) Relative current signals of the EV-iNOS level in RAW 264.7 macrophages with different phenotypes (n=3 independent experiments). Data are presented as mean ± SD. Source data are provided as a source data file.

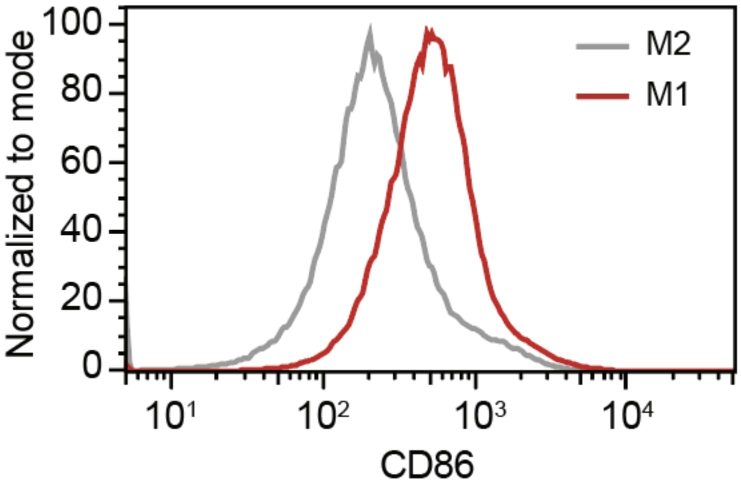

Supplementary Fig. 18 Flow cytometry analysis of CD86 expression in SC-induced macrophages with different phenotypes.

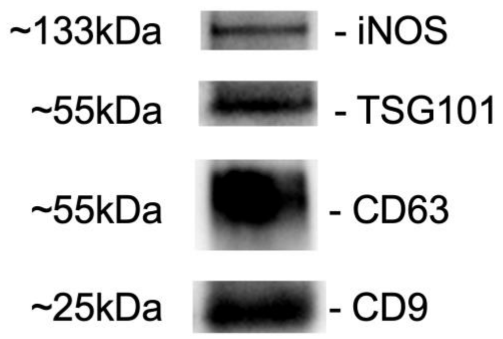

Supplementary Fig. 19 Western blot analysis of protein markers including CD9, CD63, Tsg101 and iNOS from BALF EVs collected from a patient with pneumonia. Source data are provided as a source data file.

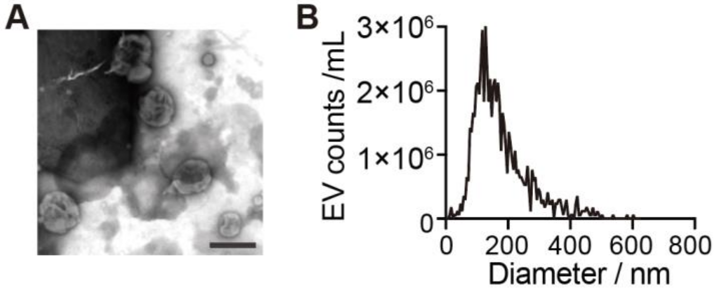

Supplementary Fig. 20 Representative TEM image (A) and particle size distribution (B) of EVs isolated from BALF of a health donor. Source data are provided as a source data file.

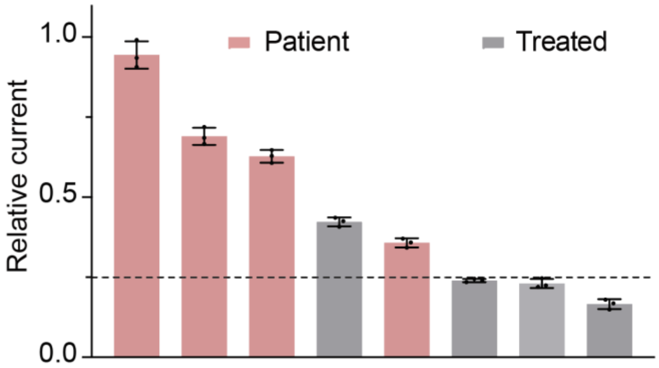

Supplementary Fig. 21 Relative current of the 4 patients before and after treatment of pneumonia (n = 3 technical replicates). Data are presented as mean ± SD. Source data are provided as a source data file.

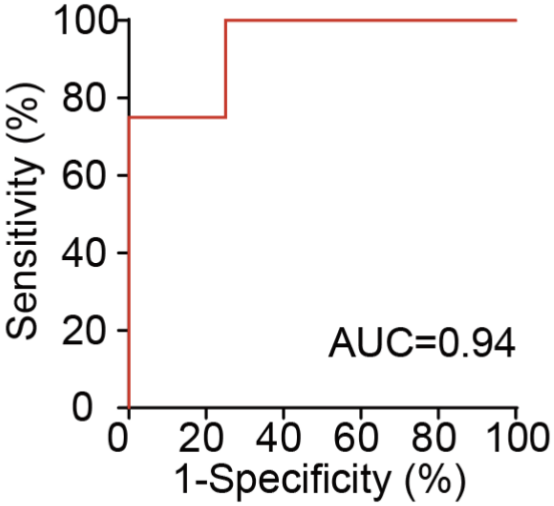

Supplementary Fig. 22 Receiver operating characteristic (ROC) curve of the metabolic EVs-based electrochemical method for the 4 patients before and after treatment of pneumonia. Source data are provided as a source data file.

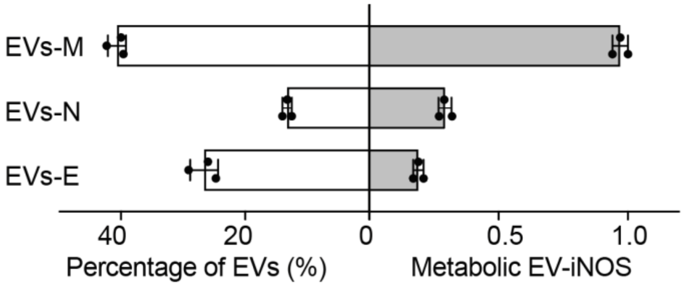

Supplementary Fig. 23 The proportion and metabolic EV-iNOS level of macrophage-derived EVs (EVs-M, $CD14^+$), neutrophil-derived EVs (EVs-N, $CD66b^+$) and epithelial cell-derived EVs (EVs-E, EpCAM+) among EVs isolated from BALF of a pneumonia patient (n=3 independent experiments). EV proportion was identified by EV-based flow cytometry and each proportion was calculated relative to total $CD63^+$ EVs (n=3 independent experiments). Data are presented as mean ± SD. Metabolic EV-iNOS level was measured following an immunobeads-based pull down assay. Source data are provided as a source data file.

Supplementary Table 1 Details for the plasmid

Gene name	mNos2-sgRNA-1
sg-seq	TCACAGCTCATCCGGTACGC
Resistance	Amp+
Vector	pSpCas9(BB)-2A-EGFP(PX458)
5'Clone site	BbsI
3'Clone site	BbsI

Supplementary Table 2 Clinical information of cohorts involved in this ACTIVITY-based pneumonia diagnosis

Characteristic	Pneumonia	Non-pneumonia	Total
**Case**	32	18	50
**Age**			
**Median**	51	37	43
**Range**	21 - 83	13 - 56	13 - 83
**Sex**			
**Male**	21 (66%)	7 (39%)	28 (56%)
**Female**	11 (44%)	11 (61%)	22 (44%)
**Serum CRP marker (mg/L)**			
**Median**	73.8	3.55	28.62
**Range**	1.72 - 280.32	0.5 – 40.15	1.72- 280.32
