# Supplementary material for: Amplifying metabolic profiling of extracellular vesicle dynamics with ACTIVITY
Source: Nat Commun. 2026 Mar 26;17:4490. doi: 10.1038/s41467-026-71030-w (PMC13187049; doi:10.1038/s41467-026-71030-w)
Supplement: Supplementary file 2 — Reporting Summary [file 41467_2026_71030_MOESM2_ESM.pdf]

nature portfolio

Corresponding author(s): Xianguang Ding

Last updated by author(s): Mar 10, 2026

Reporting Summary

Nature Portfolio wishes to improve the reproducibility of the work that we publish. This form provides structure for consistency and transparency in reporting. For further information on Nature Portfolio policies, see our Editorial Policies and the Editorial Policy Checklist.

Statistics

For all statistical analyses, confirm that the following items are present in the figure legend, table legend, main text, or Methods section.

n/a	Confirmed	
[ ]	[x]	The exact sample size (n) for each experimental group/condition, given as a discrete number and unit of measurement
[ ]	[x]	A statement on whether measurements were taken from distinct samples or whether the same sample was measured repeatedly
[ ]	[x]	The statistical test(s) used AND whether they are one- or two-sided<br>*Only common tests should be described solely by name; describe more complex techniques in the Methods section.*
[x]	[ ]	A description of all covariates tested
[ ]	[x]	A description of any assumptions or corrections, such as tests of normality and adjustment for multiple comparisons
[ ]	[x]	A full description of the statistical parameters including central tendency (e.g. means) or other basic estimates (e.g. regression coefficient) AND variation (e.g. standard deviation) or associated estimates of uncertainty (e.g. confidence intervals)
[ ]	[x]	For null hypothesis testing, the test statistic (e.g. F, t, r) with confidence intervals, effect sizes, degrees of freedom and P value noted<br>*Give P values as exact values whenever suitable.*
[x]	[ ]	For Bayesian analysis, information on the choice of priors and Markov chain Monte Carlo settings
[x]	[ ]	For hierarchical and complex designs, identification of the appropriate level for tests and full reporting of outcomes
[x]	[ ]	Estimates of effect sizes (e.g. Cohen's d, Pearson's r), indicating how they were calculated

Our web collection on statistics for biologists contains articles on many of the points above.

Software and code

Policy information about availability of computer code

Data collection	Electrochemical workstation (CHI 660E), self-designed electrochemical device (ACTIVITY), transmission electron microscope (HITACHI, HT7700), UV-Vis spectrophotometer (UV-2600, Shimadzu), X-ray photoelectron spectrometer (PHI 5000 VersaProbe, Ulvac-Phi), nanoparticle tracking analysis (NTA, Particle Metrix ZetaView), flow cytometer (BD Accuri C6), multi-plate reader (Infinite 200 PRO).
Data analysis	General data were analyzed by GraphPad prism 10.

For manuscripts utilizing custom algorithms or software that are central to the research but not yet described in published literature, software must be made available to editors and reviewers. We strongly encourage code deposition in a community repository (e.g. GitHub). See the Nature Portfolio guidelines for submitting code & software for further information.

Data

Policy information about availability of data

All manuscripts must include a data availability statement. This statement should provide the following information, where applicable:

- Accession codes, unique identifiers, or web links for publicly available datasets
- A description of any restrictions on data availability
- For clinical datasets or third party data, please ensure that the statement adheres to our policy

All the main data supporting the results are available within the main text and supplementary information.

Research involving human participants, their data, or biological material

Policy information about studies with human participants or human data. See also policy information about sex, gender (identity/presentation), and sexual orientation and race, ethnicity and racism.

Reporting on sex and gender	No selection bias.
Reporting on race, ethnicity, or other socially relevant groupings	No analysis based on ethnicity, race, or other socially relevant groupings was conducted in the current study.
Population characteristics	The current study involved bronchoalveolar lavage fluid (BALF) samples obtained with informed consent from patients with pneumonia and healthy controls, with a median age of 43.
Recruitment	Participants were recruited on standard clinical diagnosis references without patients' prior knowledge of the trial. Thus, there is no self-selection bias. Signed informed consent was obtained from patients.
Ethics oversight	Ethical Committee of the First Affiliated Hospital of Soochow University (approval no. 2023455) together with the informed consent from all patients. Participants' personal data were sterilized and were not available to the investigators.

Note that full information on the approval of the study protocol must also be provided in the manuscript.

Field-specific reporting

Please select the one below that is the best fit for your research. If you are not sure, read the appropriate sections before making your selection.

[x] Life sciences   [ ] Behavioural & social sciences   [ ] Ecological, evolutionary & environmental sciences

For a reference copy of the document with all sections, see nature.com/documents/nr-reporting-summary-flat.pdf

Life sciences study design

All studies must disclose on these points even when the disclosure is negative.

Sample size	Sample size were provided in the figure caption for each experiment and reasonable sample sizes were chosen to ensure they are sufficient for statistical comparison between different groups.
Data exclusions	No samples were excluded.
Replication	Experiment were repeated and experimental findings were reproducible. Details of experimental replicates were givens in the figure legends.
Randomization	All samples were randomly allocated into experimental groups.
Blinding	Except for device construction, all other experiments were blinded to group allocation during data collection and analysis.

Reporting for specific materials, systems and methods

We require information from authors about some types of materials, experimental systems and methods used in many studies. Here, indicate whether each material, system or method listed is relevant to your study. If you are not sure if a list item applies to your research, read the appropriate section before selecting a response.

Materials & experimental systems

n/a	Involved in the study	
[ ]	[x]	Antibodies
[ ]	[x]	Eukaryotic cell lines
[x]	[ ]	Palaeontology and archaeology
[ ]	[x]	Animals and other organisms
[x]	[ ]	Clinical data
[x]	[ ]	Dual use research of concern
[x]	[ ]	Plants

Methods

n/a	Involved in the study	
[x]	[ ]	ChIP-seq
[ ]	[x]	Flow cytometry
[x]	[ ]	MRI-based neuroimaging

Antibodies

Antibodies used	All the antibodies information can be found in Methods section and the antibodies were diluted and used following the manufacture's instructions.
Validation	All primary antibodies were purchased from the supplier, and the validation of all the antibodies can be searched on the following

websites:
anti-iNOS (ab178945): https://www.abcam.cn/products/primary-antibodies/inos-antibody-epr16635-ab178945
anti-CD63 (ab315108): https://www.abcam.cn/products/primary-antibodies/cd63-antibody-rm1095-ab315108
anti-CD9 (ET1601-9): https://huabio.cn/products/CD9-antibody-ET1601-9
anti-Tsg101 (ET1701-59): https://huabio.cn/products/TSG101-antibody-ET1701-59
APC-anti-mouse-CD86 (105012): http://www.biolegend.com/en-us/products/apc-anti-mouse-cd86-antibody-2896
PE-anti-mouse-CD206 (141706): http://www.biolegend.com/en-us/products/pe-anti-mouse-cd206-mmr-antibody-7424
APC-anti-human-CD86 (374208): https://www.biolegend.com/en-us/products/apc-anti-human-cd86-antibody-15297

Eukaryotic cell lines

Policy information about cell lines and Sex and Gender in Research

Cell line source(s)	RAW 264.7 cells (No.: TIB-71) and SC cells (No.: CBP61294) were obtained from American Type Culture Collection and Cobioer, respectively.
Authentication	Identification of the cell line was checked by morphology and growth characteristics according to the manufacturer's instructions and was used without any modification.
Mycoplasma contamination	All cell lines were tested negative for mycoplasma contamination. No mycoplasma contamination was found
Commonly misidentified lines (See ICLAC register)	No commonly misidentified cell lines were used.

Animals and other research organisms

Policy information about studies involving animals; ARRIVE guidelines recommended for reporting animal research, and Sex and Gender in Research

Laboratory animals	6-8 weeks, female, Balb/c mice were used. All mice were housed at room temperature (18-24°C) with relative humidity (40-60%) and a 12 h day-night cycle.
Wild animals	No wild animals were used.
Reporting on sex	No selection bias.
Field-collected samples	No field-collected samples were used.
Ethics oversight	All animal experiments were carried out follow the ethical guidelines and approved by the Committee for Animal Research of Nanjing University of Posts and Telecommunications (approval no. 2022012).

Note that full information on the approval of the study protocol must also be provided in the manuscript.

Plants

Seed stocks	N/A
Novel plant genotypes	N/A
Authentication	N/A

Flow Cytometry

Plots

Confirm that:

- [x] The axis labels state the marker and fluorochrome used (e.g. CD4-FITC).
- [x] The axis scales are clearly visible. Include numbers along axes only for bottom left plot of group (a 'group' is an analysis of identical markers).
- [x] All plots are contour plots with outliers or pseudocolor plots.
- [x] A numerical value for number of cells or percentage (with statistics) is provided.

Methodology

Sample preparation	Cultured cells were first maintained in a 6-well plate. The culture medium was then replaced with freshly prepared medium containing 1 μg/m fluorescence antibodies, and the cells were incubated for 30 minutes at 37°C. After washing with PBS to remove unbound antibodies, cells were analyzed by flow cytometry.
Instrument	Flow cytometry (BD Accuri C6)
Software	FlowJo v10.8.1
Cell population abundance	10^9-10^10 cells/mL
Gating strategy	Cell were gated based on size and granularity of forward and side scatter and further analyzed for specific fluorescence.

☒ Tick this box to confirm that a figure exemplifying the gating strategy is provided in the Supplementary Information.
